# Substrate and strain alter the muscle-derived mesenchymal stem cell secretome to promote myogenesis

**DOI:** 10.1186/scrt463

**Published:** 2014-06-06

**Authors:** Michael De Lisio, Tor Jensen, Richard A Sukiennik, Heather D Huntsman, Marni D Boppart

**Affiliations:** 1 Department of Kinesiology and Community Health, University of Illinois, Urbana, IL, USA; 2 Beckman Institute for Advanced Science and Technology, University of Illinois, 405 N. Mathews Avenue, MC-251, Urbana, IL 61801, USA; 3 Division of Biomedical Sciences, University of Illinois, Urbana, IL, USA

## Abstract

**Introduction:**

Mesenchymal stem cells (MSCs) reside in a variety of tissues and provide a stromal role in regulating progenitor cell function. Current studies focus on identifying the specific factors in the niche that can alter the MSC secretome, ultimately determining the effectiveness and timing of tissue repair. The purpose of the present study was to evaluate the extent to which substrate and mechanical strain simultaneously regulate MSC quantity, gene expression, and secretome.

**Methods:**

MSCs (Sca-1^+^CD45^-^) isolated from murine skeletal muscle (muscle-derived MSCs, or mMSCs) via fluorescence-activated cell sorting were seeded onto laminin (LAM)- or collagen type 1 (COL)-coated membranes and exposed to a single bout of mechanical strain (10%, 1 Hz, 5 hours).

**Results:**

mMSC proliferation was not directly affected by substrate or strain; however, gene expression of growth and inflammatory factors and extracellular matrix (ECM) proteins was downregulated in mMSCs grown on COL in a manner independent of strain. Focal adhesion kinase (FAK) may be involved in substrate regulation of mMSC secretome as FAK phosphorylation was significantly elevated 24 hours post-strain in mMSCs plated on LAM but not COL (*P* <0.05). Conditioned media (CM) from mMSCs exposed to both LAM and strain increased myoblast quantity 5.6-fold 24 hours post-treatment compared with myoblasts treated with serum-free media (*P* <0.05). This response was delayed in myoblasts treated with CM from mMSCs grown on COL.

**Conclusions:**

Here, we demonstrate that exposure to COL, the primary ECM component associated with tissue fibrosis, downregulates genes associated with growth and inflammation in mMSCs and delays the ability for mMSCs to stimulate myoblast proliferation.

## Introduction

Mesenchymal stem/stromal cells (MSCs) are a pluripotent population of cells that reside in a variety of tissues throughout the body. These cells are defined by their capacity for multi-lineage differentiation, including chondrogenesis, osteogenesis, and adipogenesis [1]. Owing to their multi-lineage potential, immune-privileged nature, relative ease of isolation, and ability to be expanded in culture, MSCs have received much attention for their potential use in cell therapy [2]. Recently, it was suggested that the primary mechanism by which MSCs contribute to tissue repair is indirect via secretion of factors that stimulate native tissue repair processes or tissue-resident stem cells [3]. It was suggested that the MSC secretome is strongly regulated by the local microenvironment [3]. For example, hypoxia can stimulate MSC secretion of vascular endothelial growth factor (VEGF) and interleukin-6 (IL-6) [4], and factors released from MSCs can reverse degenerative processes in a variety of tissues, including heart [3], brain [5], the hematopoietic system [6], and skeletal muscle [7]. For these reasons, MSCs provide an exciting cell population for therapy, and defining cell culture conditions that allow optimal MSC expansion and function prior to transplantation may be an effective strategy to enhance their efficacy.

Mesenchymal progenitor cells have been identified in skeletal muscle that directly or indirectly contributes to repair in response to injury [7]. These multi-potent stem cells have been isolated by using unique cell surface markers and thus classified as side population cells [8,9], pericytes [10], muscle-derived stem cells [11], muscle-derived MSCs (mMSCs) [12,13], fibro/adipogenic progenitors [14], and PW1^+^ interstitial cells [15], and some degree of overlap likely exists between these cell populations. Whereas some of these cells can become myogenic, the majority have limited capacity for myogenic differentiation and are primed to secrete factors essential for indirect repair of skeletal muscle. We recently defined a population of MSCs, identified by expression of stem cell antigen 1 (Sca-1^+^) and lack of expression of a hematopoietic cell surface marker (CD45^-^), that is capable of osteogenic, chondrogenic, and adipogenic differentiation and accumulate in skeletal *m*uscle (*m*MSCs) after eccentric exercise in an α7-integrin-dependent manner [12]. We also recently demonstrated that Sca-1^+^CD45^-^ mMSCs are involved in muscle repair after injury [12], perhaps through secretion of paracrine factors [16]. Currently, minimal information exists regarding the cues in the environment that regulate MSC paracrine factor synthesis and secretion [16].

The MSC niche is complex, and composition of the MSC niche in each tissue type is defined by a unique combination of growth/inflammatory factors, extracellular matrix (ECM) components, other cell types, and topography [17]. In skeletal muscle, the extracellular environment consists of a variety of matrix proteins, including but not limited to collagen type 1 (COL) and laminin (LAM) [18]. COL is the primary collagen isoform in the perimysium and accumulates during muscle fibrosis [18] that accompanies disease, injury, and aging [19,20]. The resulting fibrosis causes dysregulation of muscle-derived stem cells which may be a major factor inhibiting muscle regeneration and repair [19,21]. Because the musculoskeletal system is highly dynamic and responsive to movement, mechanical strain may largely intersect and alter the MSC response to COL or LAM or both [22]. Some information exists regarding MSC mechanosensory signaling and lineage specification. For example, MSCs derived from bone marrow are directed toward an osteogenic fate when subjected to multiple bouts of biaxial strain [23-25] and this may be dependent on the activation of focal adhesion kinase (FAK) [10]. In addition, mMSCs secrete a variety of growth factors in response to biaxial strain in the presence of LAM [16]. However, the full extent to which MSCs residing in skeletal muscle are responsive to mechanical strain in the context of substrate has yet to be determined. This information is essential for elucidating a role for mMSCs in whole tissue health and designing cell-based therapies that can most effectively combat skeletal muscle disease and disabilities.

The purpose of the present investigation was to characterize the mMSC response to mechanical strain in the context of substrate. We hypothesized that mechanical strain would increase mMSC quantity or gene expression of factors that would promote skeletal muscle regeneration (or both) while exposure to COL would inhibit this response.

## Materials and methods

### α7 Integrin transgenic mice

Mice created to overexpress α7 integrin specifically in skeletal muscle (MCK-α7BX2), referred to as α7Tg mice, were produced and bred in-house [26]. Overexpression of α7 integrin BX2 subunit was determined by polymerase chain reaction (PCR) analysis (primers: MCK1, 5′-CAAGCTGCACGCCTGGGTCC-3′ and AATII, 5′-GGCACCCATGACGTCCAGATTGAAG-3′) as previously described [27]. Mice were maintained on a 12-hour light/dark cycle and were provided food and water *ad libitum*. All protocols were approved by the Institutional Animal Care and Use Committee of the University of Illinois at Urbana-Champaign.

### Muscle-derived mesenchymal stem/stromal cell isolation

mMSCs were isolated as previously described [12,16]. Briefly, 5- to 6-week-old α7Tg mice were sacrificed by carbon dioxide (CO_2_) asphyxiation, and gastrocnemius-soleus complexes were resected and minced into a fine paste. Minced muscle tissue was enzymatically digested in phosphate-buffered saline containing 0.2% collagenase Type 2 (Worthington, Biochemical Corp., Lakewood, NJ, USA), 60 U/mL DNase (Sigma-Aldrich, St. Louis, MO, USA), and 2.5 mM Ca_2_Cl_2_ at 37°C for 45 minutes. Muscle slurries were mixed with Hank’s Balanced Salt Solution containing 20% fetal bovine serum (FBS) and 0.1% penicillin/streptomycin to stop enzymatic digestion and were passed through a 70-μm filter. Cells were blocked in Fc-receptor block (CD16/CD32; eBioscience, San Diego, CA, USA) for 10 minutes on ice and then stained with Sca-1-PE and CD45-APC (eBioscience). After washing, cells were passed through a 40-μm filter prior to sorting on an iCyt Reflection System (Champaign, IL, USA). Unstained and single-stained controls were used to establish gates. Sorted cells were seeded onto plastic cell culture dishes at a density of 2.5 × 10^4^ cells per cm^2^ in growth media (Dulbecco’s modified Eagle’s medium [DMEM]/10% FBS/5 μg/mL gentamicin). After 3 days of culture at 37°C and 5% CO_2_, non-adherent cells were removed.

### *In vitro* strain protocol and assessment of muscle-derived mesenchymal stem/stromal cell quantity

Passage-1 or -2 mMSCs were seeded at equal density on either laminin (YIGSR; LAM)- or collagen type 1 (COL)-coated Flexcell plates (Flexcell International Corporation, McKeesport, PA, USA). Cells were allowed to adhere and expand for 12 to 72 hours in growth media until they were approximately 80% confluent. Cells were strained according to our previously published protocol that has been shown to alter myogenic gene expression in mMSCs [12] and the mMSC secretome in a manner that supported arteriogenesis [16]. Briefly, cells were exposed to 10% biaxial strain at a frequency of 1 Hz for 5 hours using an FX-4000 Flexercell Tension System (Flexcell International Corporation). Non-strained cells were maintained in the same manner but not exposed to strain. Four experimental conditions were tested: (1) LAM/No Strain (NSL), (2) LAM/Strain (SL), (3) COL/No Strain (NSC), and (4) COL/Strain (SC). For conditioned media (CM) experiments, growth media were replaced with serum-free media (SFM) (DMEM/5 μg/mL gentamicin) prior to the strain protocol, collected at 24 hours after the initiation of strain, and stored at -80°C until use. mMSCs were trypsinized and quantified by using a hemocytometer 48 hours after strain. All images were obtained by using a Zeiss AxioCam digital camera and Axiovision software at 20× magnification (Zeiss, Thornwood, NY, USA).

### Gene expression analysis

Three hours after stretch, mMSCs were collected by cell scraping and were centrifuged, and the pellet was frozen at -80°C. Isolation of total RNA was conducted by using the PureLink RNA mini kit (Ambion, a brand of Life Technologies, Carlsbad, CA, USA) in accordance with the instructions of the manufacturer. The RNA was quantified spectrophotometrically via the Synergy H1 Hybrid Multi-Mode Microplate Reader (Biotek, Winooski, VT, USA). Reverse transcription of RNA to cDNA was completed by using a High Capacity cDNA RT Kit (Applied Biosystems, Grand Island, NY). Quantitative real-time polymerase chain reaction (qPCR) was performed either by using SYBR Green/Rox master mix (SuperArray Biosciences, Frederick, MD, USA) with previously published primer sequences or by using pre-designed Taqman primers and probes validated by Applied Biosystems (Foster City, CA, USA) (Tables 1 and 2). qPCR was performed by using the ABI 7900HT Fast Real Time PCR System (Applied Biosystems) under standard conditions and analyzed by using SDS 2.4 Software (Applied Biosystems). Dissociation curve analysis was performed for SYBR Green reactions to confirm primer specificity. Changes in gene expression relative to LAM/NS were calculated by using the 2^-ΔΔCT^ method [28] with GAPDH or L32 as housekeeping genes.

**Table 1:** Primer pairs for SYBR Green analysis

**Gene**	**Forward**	**Reverse**
*Myf5*	TGAAGGATGGACATGACGGACG	TTGTGTGCTCCGAAGGCTGCTA
*MyoD*	TACCCAAGGTGGAGATCCTG	CATCATGCCATCAGAGCAGT
*Myogenin*	GAGACATCCCCCTATTTCTACCA	GCTCAGTCCGCTCATAGCC
*Pax7*	CTGGATGAGGGCTCAGATGT	GGTTAGCTCCTGCCTGCTTA
*L32*	TCCACAATGTCAAGGAGCTG	ACTCATTTTCTTCGCTGCGT
*COL1α2*	GCAGGTTCACCTACTCTGTCCT	CTTGCCCCATTCATTTGTCT
*Laminin α1*	CAGCGCCAATGCTACCTGT	GGATTCGTACTGTTACCGTCACA
*Laminin α2*	TCCCAAGCGCATCAACAGAG	CAGTACATCTCGGGTCCTTTTTC
*GM-CSF*	ACCACCTATGCGGATTTCAT	TCATTACGCAGGCACAAAAG
*HGF*	ATGTGGGGGACCAAACTTCTG	GGATGGCGACATGAAGCAG

**Table 2:** Primers and probes for Taqman analysis

**Gene**	**Assay ID**	**Gene ID**
*VEGFa*	Mm01281449_m1	22339
*IL-6*	Mm00446190_m1	16193
*TGF-β1*	Mm01178820_m1	21803
*TNF-α*	Mm00443258_m1	21926
*COL1α1*	Mm00801666_g1	12842
*GAPDH*	Mm03302249_g1	14433

### Western blotting

Proteins were extracted directly from cell culture plates in ice-cold lysis buffer consisting of 50 mM Tris-HCl, 150 nM NaCl, 1 mM PMSF, 1% NP-40, 0.5% sodium deoxycholate, and 0.1% SDS with protease and phosphatase inhibitors (Roche, Indianapolis, IN, USA). Samples were incubated in lysis buffer for 45 minutes at 4°C, and supernatants were collected for determination of protein concentrations by Bradford assay. Equal amounts of protein, as confirmed by ponceau S, in 4X Laemlli buffer were separated by SDS-PAGE and transferred to nitrocellulose membranes. After blocking with 5% non-fat dry milk or 5% bovine serum albumin, membranes were incubated in the following primary antibodies: phospho-mTOR (Ser2448), mTOR, p70 S6 Kinase, phospho-p70 S6 Kinase (Thr389), phospho-FAK (Tyr 397), phospho-NF-κB p65 (Ser 536) (Cell Signaling Technology, Boston, MA, USA), FAK (Santa Cruz Biotechnology, Santa Cruz, CA, USA), or NF-κB p65 (eBioscience) overnight at 4°C. Horseradish peroxidase-conjugated anti-rabbit secondary antibody (Jackson ImmunoResearch Laboratories, Inc., West Grove, PA, USA) was applied for 1 hour at room temperature. Proteins were detected with SuperSignal West Pico Chemiluminescent Substrate (Pierce, Rockford, IL, USA) by using a Bio-Rad ChemiDoc XRS system (Bio-Rad Laboratories, Hercules, CA, USA). After detection of phosphorylated proteins, membranes were stripped with Restore Western Blot Stripping Buffer (Thermo Fisher Scientific, Waltham, MA, USA) prior to probing for total protein. Protein content was quantified by using Quantity One software (Bio-Rad Laboratories) with background subtracted. Any samples that did not give values above background were omitted from analysis. For each protein, phosphorylated protein content was expressed relative to total protein content and normalized to the average of LAM/NS to obtain a fold change.

### Myoblast and myotube culture experiments

Littermates of α7Tg mice that did not overexpress the transgene were used for primary myoblast isolation. Myoblasts were isolated from neonatal mice by using a sequential plating technique as previously described [29], except plates were coated with gelatin instead of collagen as we have found that gelatin increases the efficiency of isolation. Primary myoblasts and C2C12 cells (ATCC, Manassas, VA, USA) were maintained at 37°C in 5% CO_2_, expanded in growth media (DMEM/20% FBS/1% penicillin-streptomycin), and differentiated in differentiation media (DMEM/2% horse serum/1% penicillin-streptomycin). For CM experiments, CM was quickly thawed, centrifuged at 400 *g* for 5 minutes at 4°C to remove any remaining cells, and added to primary myoblasts seeded at equal density or myotubes formed from C2C12 myoblasts. Myoblasts or myotubes treated with SFM and either growth or differentiation media served as negative and positive controls. Myotubes were harvested 3 hours after treatment with CM for evaluation of hypertrophic signaling. Myoblast quantity was analyzed at 24 and 48 hours post-treatment with CM via the MTT (3-[4,5-dimethylthiazol-2-yl]-2,5-diphenyltetrazolium bromide; thiazolyl blue)-based Cell Growth Determination Kit (Sigma-Aldrich) in accordance with the instructions of the manufacturer, and values were expressed as a fold change relative to myoblasts treated with SFM for the same amount of time.

### Statistical analysis

Data are presented as mean ± standard error of the mean. Two-factor analysis of variance (substrate × strain) was conducted followed by Tukey *post hoc* test if a significant difference was detected. All statistical analyses were conducted by using SigmaPlot 12.5 (Systat Software, Inc., Chicago, IL, USA). Differences were considered significant at a *P* value of less than 0.05.

## Results

### Exposure to different substrates or strain does not alter muscle-derived mesenchymal stem/stromal cell quantity but alters myogenic gene expression

**Figure 1:**
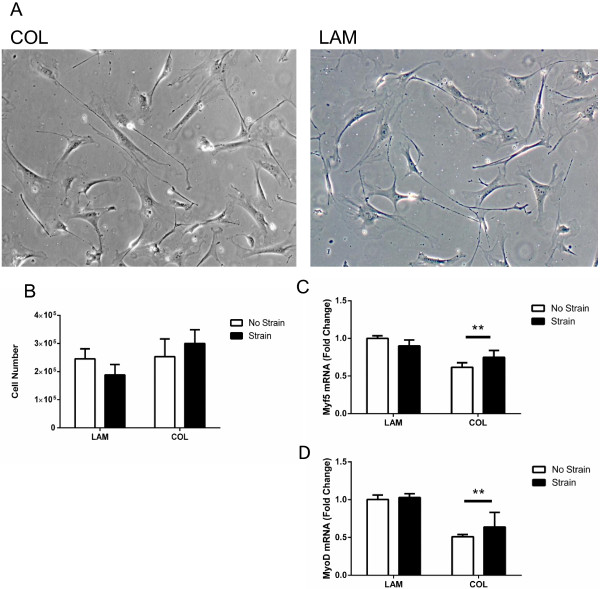
**Muscle-derived mesenchymal stem/stromal cell (mMSC) appearance, proliferation, and myogenic gene expression.** mMSCs were plated on laminin (LAM) or collagen (COL) and were either exposed to mechanical strain or remained unstrained. Representative images of mMSCs exposed to the various substrates **(A)**. mMSC quantity as determined by hemocytometer 48 hours post-strain with non-strained cells serving as controls (n = 6 per group) **(B)**. Gene expression of the myogenic regulatory factors Myf5 **(C)**, MyoD **(D)**, and Myogenin **(E)** 3 hours post-strain (n = 3 per group). Pax7 mRNA was not reliably detected in any group. Fold change is relative to LAM no strain. Data are presented as mean ± standard error of the mean. **P <0.01 versus LAM (main effect for substrate).

mMSCs displayed their characteristic fibroblast-like appearance with numerous projections spreading out from the cell body on both COL and LAM (Figure 1A). Neither substrate nor strain influenced the quantity of mMSCs at 48 hours (Figure 1B). Gene expressions of the myogenic regulatory factors (MRFs) Myf5 (*P* <0.01), MyoD (*P* <0.01), and Myogenin (*P* <0.05) were all significantly downregulated in mMSCs grown on COL (Figure 1C-E). The myoblast-specific transcription factor Pax7 was not reliably detected in any samples analyzed (data not shown).

### Muscle-derived mesenchymal stem/stromal cell gene expression is downregulated on collagen 1

**Figure 2:**
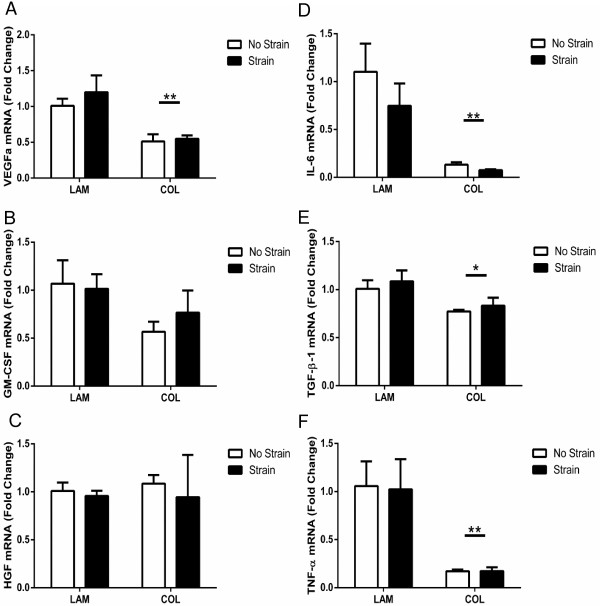
**Muscle-derived mesenchymal stem/stromal cell (mMSC) growth and inflammatory factor gene expression.** mMSCs were plated on laminin (LAM) or collagen (COL) and were either exposed to mechanical strain or remained unstrained. mRNA for vascular endothelial growth factor a (VEGFa) **(A)**, granulocyte-macrophage colony-stimulating factor (GM-CSF) **(B)**, hepatocyte growth factor (HGF) **(C)**, interleukin-6 (IL-6) **(D)**, transforming growth factor-beta 1 (TGF-β1) **(E)**, and tumor necrosis factor-alpha (TNF-α) **(F)** was analyzed 3 hours after strain and expressed relative to LAM no strain (n = 3 per group). Data are presented as mean ± standard error of the mean. *P <0.05; **P <0.01 versus LAM.

**Figure 3:**
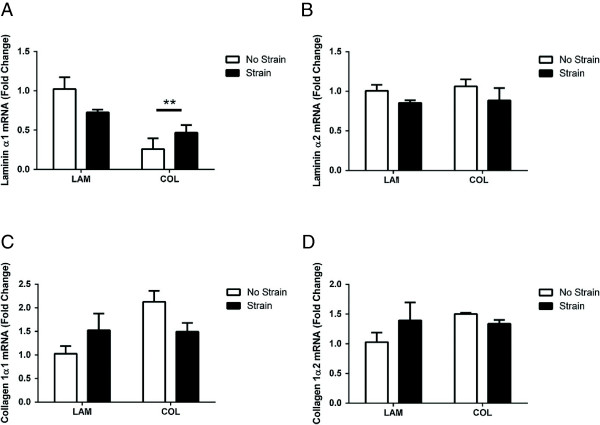
**Muscle-derived mesenchymal stem/stromal cell (mMSC) extracellular matrix gene expression.** mMSCs were plated on laminin (LAM) or collagen (COL) and were either exposed to mechanical strain or remained unstrained. mRNA for laminin α1 **(A)**, laminin α2 **(B)**, collagen 1α1 **(C)**, and collagen 1α2 **(D)** was analyzed 3 hours after strain and expressed relative to LAM no strain (n = 3 per group). Data are presented as mean ± standard error of the mean. **P <0.01 versus LAM.

Growth factor gene expression was generally downregulated in mMSCs grown on COL with a significant decrease in the expression of VEGFa (Figure 2A; *P* <0.01), a trend toward a decrease in expression of granulocyte-macrophage colony-stimulating factor (GM-CSF) (Figure 2B; *P* = 0.087), and no change in hepatocyte growth factor (HGF) expression (Figure 2C). Similarly, gene expression of the inflammatory cytokines IL-6 (*P* <0.01), transforming growth factor-beta 1 (TGF-β1) (*P* <0.05), and tumor necrosis factor-alpha (TNF-α) (*P* <0.01) was significantly decreased in mMSCs grown on COL (Figure 2D-F). The expression of growth or inflammatory factors was not influenced by strain (Figure 2A-F). Laminin α1 gene expression was significantly decreased (Figure 3A; *P* <0.01) in mMSCs on COL, whereas laminin α2 was not altered (Figure 3B). There was a strong trend for an interaction (*P* = 0.050) between substrate and strain for collagen 1α1 (Figure 3C) with no differences in expression of collagen 1α2.

### Substrate and stretch alter the activation of focal adhesion kinase but not nuclear factor-kappa B

**Figure 4:**
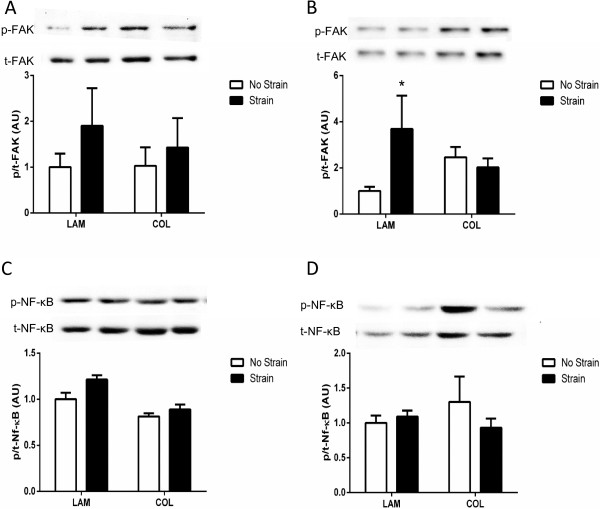
**Signaling in muscle-derived mesenchymal stem/stromal cell (mMSCs).** mMSCs were plated on laminin (LAM) or collagen (COL) and were either exposed to mechanical strain or remained unstrained. mMSCs were harvested and activation of focal adhesion kinase (FAK) was determined at 3 hours **(A)** and 24 hours **(B)** post-strain. Activation of nuclear factor-kappa B (NF-κB) was also analyzed at 3 hours **(C)** and 24 hours **(D)** post-strain. Representative images are shown above each respective blot. Data are presented as mean ± standard error of the mean. *P <0.05 versus No Strain/LAM. AU, arbitrary units.

FAK is an intracellular signaling molecule that responds to various factors in the cellular microenvironment and may facilitate the process of mechanotransduction [30]. Levels of phosphorylated FAK were not altered on either substrate 3 hours post-strain (Figure 4A). At 24 hours post-strain, FAK phosphorylation was significantly increased in mMSCs strained on LAM (Figure 4B; *P* <0.05), whereas total levels of FAK were increased on COL (data not shown). Neither NF-κB phosphorylation nor levels of total protein were altered by substrate or strain at either time point (Figure 4C-D).

**Figure 5:**
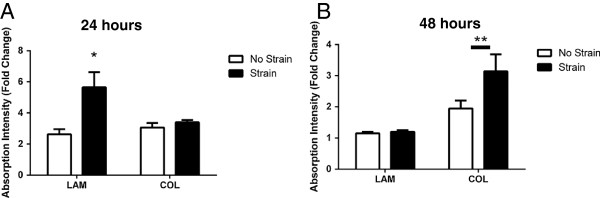
**Myoblast proliferation after treatment with muscle-derived mesenchymal stem/stromal cell (mMSC) conditioned media.** mMSCs were plated on laminin (LAM) or collagen (COL) and were either exposed to mechanical strain or remained unstrained. The quantity of primary myoblasts treated with conditioned media for 24 hours **(A)** or 48 hours **(B)** was determined by MTT assay. Data are presented as mean ± standard error of the mean of fold change relative to myoblasts treated with serum-free media for the same amount of time. *P <0.05 versus Lam/No Strain. **P <0.01 versus LAM with n = 6 per group for the 24-hour time point and n = 7 per group for the 48-hour time point. MTT, 3-[4,5-dimethylthiazol-2-yl]-2,5-diphenyltetrazolium bromide.

### The muscle-derived mesenchymal stem/stromal cell secretome increases myoblast quantity but does not influence hypertrophic signaling in myotubes

**Figure 6:**
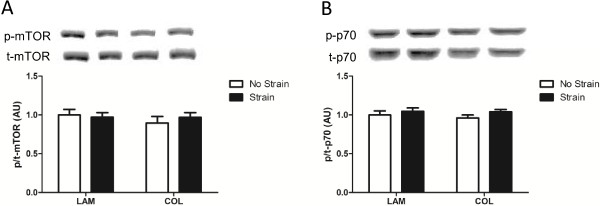
**Hypertrophic signaling in C2C12-derived myotubes exposed to muscle-derived mesenchymal stem/stromal cell (mMSC) conditioned media.** mMSCs were plated on laminin (LAM) or collagen (COL) and were either exposed to mechanical strain or remained unstrained. C2C12-derived myotubes treated with conditioned media for 3 hours were harvested and analyzed for activation of mammalian target of rapamycin (mTOR) **(A)** and p70 **(B)**. Representative images are shown above each respective blot. Data are presented as mean ± standard error of the mean. AU, arbitrary units.

CM were collected from mMSCs not strained on LAM (NSL), strained on LAM (SL), not strained on COL (NSC), and strained on COL (SC) and used to treat C2C12-derived myotubes or primary myoblasts. CM from SL increased the quantity of primary myoblasts after 24 hours of treatment as compared with NSL and SC (Figure 5A; *P* <0.01 and *P* <0.001, respectively). After 48 hours of treatment, primary myoblasts treated with CM from mMSCs grown on COL were significantly increased in quantity (Figure 5B; *P* <0.001 main effect for COL) and a strong trend was observed for increased quantity of primary myoblasts when exposed to CM from strained mMSCs (*P* = 0.050 for strain). In myotubes, activation of two signaling molecules involved in the initiation of protein synthesis, mTOR and p70 S6 Kinase, was not altered at 3 hours when exposed to CM from any of the above conditions (Figure 6A and B).

## Discussion

In the present investigation, we demonstrate that mMSCs are responsive to different substrates in their environment and, to a lesser extent, mechanical strain. COL, an ECM protein that accumulates in fibrotic conditions, resulted in decreased expression of a wide variety of genes, including MRFs, growth factors, cytokines, and ECM proteins in mMSCs. These results suggest that mMSC interaction with collagen decreases their potential for myogenic differentiation and decreases their production of growth and inflammatory factors involved acutely in muscle repair or chronically in muscle degeneration. Our CM experiments demonstrated that media from mMSCs strained on LAM enhanced myoblast proliferation at 24 hours while this response was delayed to 48 hours in myoblasts treated with CM from mMSCs grown on COL. Overall, these data suggest that collagen accumulation in fibrotic conditions may impair the myogenic response by inhibiting mMSC function.

mMSCs exist in limited quantities *in vivo*; therefore, identifying factors that promote expansion or enhance their function would increase the clinical utility of these cells. In agreement with previous findings in primary myoblasts [31], we did not observe any differences in mMSC quantity when grown on LAM or COL. Furthermore, strain did not enhance mMSC proliferation on either substrate. mMSCs may contribute to muscle regeneration or repair by direct differentiation or fusion with existing muscle fibers or indirectly via the promotion of satellite cell activation and differentiation [32]. Expression of the MRFs Myf5, MyoD, and myogenin was downregulated on COL, suggesting that direct differentiation toward the myogenic lineage was suppressed. This is in agreement with a previous study that examined the effects of different substrates on expression of Myf5 as well as myotube formation of a mixed population of primary myoblasts and demonstrated an increase in both of these parameters in cells plated on laminin [31]. Furthermore, it has been suggested that osteogenic differentiation of MSCs is promoted on collagen 1 [33,34], supporting the notion that COL inhibits myogenic differentiation. Pax7 expression was also not reliably detected in any group and this is in agreement with a previous study demonstrating low Pax7 expression in MSCs [35] and with a previous study from our group [12]. Together, these data suggest that LAM maintains the expression of genes required for myogenesis, while COL downregulates these genes and promotes differentiation along other lineages. The pro-myogenic response on LAM may be related to VEGF signaling as inhibition of VEGF induction in response to mechanical strain decreased the myogenic potential of muscle-derived stem cells (MDSCs) [36], and VEGF expression was significantly downregulated in mMSCs on COL.

Similar to expression of the MRFs and VEGF, gene expression of several inflammatory factors was downregulated on COL. The effects of inflammatory factors on myogenesis are complex with chronic and acute exposures having differential effects. The role of IL-6 on myogenesis has been extensively evaluated with ablation of IL-6 linked to impaired muscle hypertrophy [37], and myoblasts treated with IL-6 *in vitro* displayed increased differentiation [38]. In addition, IL-6 localizes to human satellite cells from young individuals acutely following eccentric damage [39,40], while this response is dysfunctional with age and may contribute to decreased muscle repair [41]. Acutely, increases in IL-6 and TNF-α can promote myogenesis as TNF-α increased satellite cell proliferation *in vivo* and *in vitro*[42]. The pattern of TNF-α expression mirrors that of IL-6, suggesting that these two factors are similarly regulated. On the other hand, chronic exposure to inflammatory cytokines, including IL-6 and TNF-α, has been shown to have detrimental effects on myogenesis [43]. Elevated systemic levels of IL-6 have been associated with muscle wasting in cancer [44] and aging [45]. The present study indicates that mMSCs are a rich source of paracrine factors and that expression of these factors is downregulated on COL. Downregulation of inflammatory factor production on COL may be a protective mechanism to inhibit chronic upregulation of these factors in fibrotic environments that would further contribute to muscle wasting. Further support for this notion is seen in the trend for a decrease (*P* = 0.087) in GM-CSF expression in mMSCs on COL. GM-CSF is involved in the initial repair response of skeletal muscle to injury or mechanical strain through the recruitment of inflammatory cells [46]. If mMSCs recognize COL as a signal for fibrosis or a repaired wound, then decreased recruitment of inflammatory mediators would be a beneficial response for inhibiting propagation of the inflammatory signal.

Aside from altering the paracrine factor composition within the muscle microenvironment, mMSCs may restructure the ECM. Differential expression of laminin α1 and collagen 1α1 was observed in mMSCs plated on different substrates. Laminin α1, a developmentally regulated, critical component of the laminin trimer [47], was downregulated in mMSCs exposed to COL. Laminin-α1β1γ1 (LM-111) has previously been used as an effective therapy in pre-clinical models for the treatment of muscular dystrophies [48-50], indicating that LAM may improve the muscle microenvironment and increase muscle repair in pathological conditions where attachment of muscle fibers to the basal lamina is impaired. The precise mechanism by which LM-111 can promote regeneration in healthy adult muscle is not currently known. Interestingly, the only mRNA in this study that displayed some regulation by mechanical strain was collagen 1α1, which exhibited a strong trend for an interaction (*P* = 0.050), increasing on LAM with strain and decreasing on COL with strain. The upregulation of collagen 1α1 in response to strain on LAM may indicate the early stages of ECM remodeling similar to what has been demonstrated with exercise [51]. The basally elevated levels of collagen 1α1 expression in mMSCs grown on COL are reminiscent of cellular dysregulation in fibrotic conditions [19,20].

A potential explanation for the decreased expression of inflammatory and ECM-related genes in mMSCs plated on collagen is that mMSCs plated on collagen recognize this collagen-rich environment as a healed wound. TGF-β1 is well known to promote wound healing acutely, whereas excess TGF-β1 can lead to fibrosis [52], potentially via the promotion of mMSC differentiation toward a myofibroblast phenotype [53]. Myofibroblasts are derived from a variety of cell types, including MSCs, and acute activation of myofibroblasts is important for tissue repair as they are responsible for secreting ECM proteins that form a new matrix for repaired or remodeled tissue [54]. In the present study, mMSCs plated on COL decreased their level of TGF-β1 expression, suggesting that their differentiation toward a myofibroblast phenotype may have been impaired. These data suggest a mechanism whereby TGF-β1 is downregulated on COL inhibiting the myofibroblast phenotype and ECM remodeling. It is important to note that chronic activation of myofibroblasts can lead to tissue fibrosis and contracture [54]. Thus, mMSCs plated on COL may recognize their environment as one that is already fully healed [52] and downregulate their expression of various repair factors, including inflammatory cytokines and ECM proteins, while plating on LAM may mimic an environment in the early stages of repair.

The alterations in mMSC gene expression in the presence of COL and LAM prompted us to investigate the capacity for the mMSC secretome to differentially impact myoblast activation and myotube growth. *In vitro* experiments were conducted to determine the full extent to which the mMSC secretome could impact these events in muscle. We did not observe any change in anabolic signaling (mTOR, p70) in myotubes treated with CM from mMSCs, suggesting that stimulation of protein synthesis is not the primary mechanism whereby mMSCs contribute to muscle repair or hypertrophy. On the other hand, we did observe differential regulation of myoblast proliferation in CM experiments. CM from mMSCs strained on LAM increased myoblast quantity 24 hours post-treatment relative to all other conditions. These differential effects on myoblast proliferation were seen despite no differences in HGF expression between mMSCs on LAM or COL with or without strain. HGF is the ligand for the c-met receptor and initiates satellite cell activation [55]. Since myoblasts in culture are already actively proliferating, HGF may not be the primary paracrine factor underlying this response. The impact of strain on myoblast proliferation was unexpected given the lack of change in gene expression in response to strain. However, the delayed increase in FAK phosphorylation at 24 hours post-strain in mMSCs exposed to LAM suggests that strain may have a long-term effect on intracellular actin remodeling or adhesion or both. We hypothesize that growth on LAM is a signal to increase the transcription of various growth/inflammatory factors and that mechanical strain is the stimulus to allow the release of these factors. Indeed, previous studies have shown that mechanical strain promotes release of growth factors from mMSCs [16] and MDSCs [36]. Differential production of paracrine factors in mMSCs plated on LAM versus COL may explain the upregulation of myoblast quantity at 24 hours when treated with LAM CM versus the delayed increase to 48 hours in myoblasts treated with COL CM as the concentration of paracrine factors in LAM CM may have been higher. Whether the delay in myoblast expansion observed with CM from COL- and strain-treated mMSCs at 48 hours is reflective of a corresponding time delay in FAK phosphorylation is not known.

In humans, NF-κB expression following eccentric contraction was recently linked to paracrine factor release, and NF-κB was localized primarily to pericytes, a population of cells similar to mMSCs [10]. Since we observed decreased inflammatory factor expression in mMSCs plated on COL in the present study, we examined the activation of NF-κB in mMSCs on LAM and COL with or without strain. We did not observe any alterations in levels of phosphorylated or total NF-κB under the conditions explored in the present investigation, suggesting that different mechanisms may be responsible for the effects observed.

A potential limitation of our study is the use of commercially available Flexcell plates pre-coated with laminin and collagen peptide. This model has been used previously in the literature to examine the effects of different substrates [31] and mechanical strain [12,16,56] on primary myoblasts and mMSCs. The data presented here provide the first step in evaluating the mMSC response to strain in the context of substrate, yet further studies are necessary to confirm and expand this information. Our lab is currently developing novel methods that will allow the evaluation of the mMSC response to strain in the presence of a wide variety of full-length substrates in the context of stiffness. These studies are essential to fully understand the role for mMSCs in tissue health and assist in the development of stem cell-based therapies.

The data presented here demonstrate that MSCs are responsive to their microenvironment and this may suggest that injecting MSCs into an inflamed or fibrotic environment could alter their secretome and impair their capacity for regeneration. Previous literature would suggest that the MSC secretome is maintained for at least a short while after transplantation into an inflamed or fibrotic environment. MSC co-transplantation with hematopoietic stem cells in a hematopoietic stem cell transplant promotes hematopoietic regeneration although donor MSCs are not detected in recipient bone marrow at late time points following transplantation [57,58]. In cardiac infarction models, MSCs promote cardiac remodeling through secretion of paracrine factors detectable 2 weeks post-transplantation [59] although the majority of MSCs die within 4 days of transplantation [60]. Together, these studies suggest that MSCs are rapidly lost following transplantation and this may represent a protective mechanism that prevents their secretome from being altered when transplanted into an inflamed or fibrotic environment. The beneficial effects of MSC therapy in these models are realized acutely after MSC transplantation that initiates a local regenerative response through release of paracrine factors.

## Conclusions

mMSCs are responsive to their environment and may participate in the detrimental changes in tissue health in fibrotic conditions. Growth on collagen 1 inhibits gene expression and alters the secretion of factors involved in myoblast proliferation or survival or both. Further studies should focus on the extent to which MSC preconditioning with laminin or mechanical strain or both can improve outcomes associated with MSC transplantation as well as determine the extent to which laminin or physical activity or both can directly recover endogenous mMSC function in diseased and aged skeletal muscle.

## Abbreviations

CM: conditioned media; CO_2_: carbon dioxide; COL: collagen; DMEM: Dulbecco’s modified Eagle’s medium; ECM: extracellular matrix; FAK: focal adhesion kinase; FBS: fetal bovine serum; GM-CSF: granulocyte-macrophage colony-stimulating factor; HGF: hepatocyte growth factor; IL-6: interleukin-6; LAM: laminin; LM-111: laminin-111; MDSC: muscle-derived stem cell; mMSC: muscle-derived mesenchymal stem/stromal cell; MRF: myogenic regulatory factor; MSC: mesenchymal stem/stromal cell; mTOR: mammalian target of rapamycin; NF-κB: nuclear factor-kappa B; NSC: collagen-No Strain; NSL: laminin-No Strain; PCR: polymerase chain reaction; qPCR: quantitative polymerase chain reaction; SC: collagen 1-Strain; SFM: serum-free media; SL: laminin-Strain; TGF-β1: transforming growth factor-beta 1; TNF-α: tumor necrosis factor-alpha; VEGF: vascular endothelial growth factor.

## Competing interests

The authors declare that they have no competing interests.

## Authors’ contributions

MD helped to design the study, write the manuscript, and complete experiments and analyzed the data. MB helped to design the study and write the manuscript. TJ, RAS, and HDH helped to complete experiments. All authors read and approved the final manuscript.
